# Effect of forest structural attributes on soil microbial diversity in mixed temperate forests

**DOI:** 10.1007/s11104-025-07907-4

**Published:** 2025-10-01

**Authors:** Devara P. Adiningrat, Andjin Siegenthaler, Michael Schlund, Tiejun Wang, Andrew K. Skidmore, Mélody Rousseau, Marco Heurich

**Affiliations:** 1 Faculty of Geo-Information Science and Earth Observation, University of Twente, Enschede, 7522 NH The Netherlands; 2 Department of National Park Monitoring and Animal Management, Bavarian Forest National Park , Freyunger Str. 2, 94481 Grafenau, Germany; 3 Department of Forestry and Wildlife Management, Inland Norway University of Applied Sciences, Oslo, Norway

**Keywords:** Bacteria, Fungi, Taxonomic richness and diversity, Community composition, Remote Sensing, eDNA

## Abstract

**Background and aims:**

Forest structures create diverse ecological niches that sustain biodiversity across various taxa. Despite their ecological importance, the relationship between forest structure and belowground biodiversity, particularly soil microbial communities (bacterial and fungal), remains underexplored. This study investigates how forest structural attributes affect soil microbial diversity and community composition in a mixed temperate forest in the Bavarian Forest National Park, Germany.

**Method:**

Forest structure variables, including stand-level attributes, structural complexity, and understory configuration, together with selected soil properties, were derived from field measurements and LiDAR data and used as predictors. Soil microbial diversity was assessed through high-throughput eDNA sequencing across 85 plots.

**Results:**

Forest structural attributes explained significant variations in soil microbial diversity, but less so for community composition. Tree size (height and diameter) had the most consistent effect on both diversity and composition, while structural complexity primarily influenced community composition.

**Conclusion:**

Forest structural attributes offer valuable insights into soil microbial diversity patterns. This study underscores the potential for integrating remote sensing and eDNA data to monitor and map belowground biodiversity on larger spatial scales.

**Supplementary Information:**

The online version contains supplementary material available at 10.1007/s11104-025-07907-4.

## Introduction

Soil microbial communities, composed primarily of bacteria and fungi, are key drivers of nutrient cycling in forest ecosystems (Baldrian 2017; Lladó et al. 2017). Bacteria are primary decomposers of labile organic matter and mediate key biogeochemical processes such as nitrogen fixation, nitrification, and denitrification (Lladó et al. 2017). Their metabolic versatility allows them to rapidly respond to environmental changes, influencing nutrient availability and soil fertility (Fierer 2017). In contrast, fungi, particularly mycorrhizal and saprotrophic species, are crucial for decomposing complex organic matter such as lignin and cellulose, facilitating carbon sequestration, immobilizing nutrients in inorganic matter, and transferring nutrients to trees (Crowther et al. 2019; Gömöryová et al. 2013; Uroz et al. 2016). Mycorrhizal fungi establish symbiotic relationships with plants by enhancing nutrient uptake, particularly phosphorus, while also contributing to soil structure (Baldrian 2017). These roles of bacteria and fungi are essential for maintaining soil fertility, supporting tree growth, and sustaining broader ecosystem functions. Largely due to these strong associations with vegetation, soil microbial communities are closely linked to aboveground processes and plant functional traits (de Vries et al. 2012). In addition to these aboveground influences, microbial diversity and composition are also strongly shaped by soil properties such as pH, moisture, organic matter content, and nutrient availability (Fierer and Jackson 2006; Jiang et al. 2021; Kopáček et al. 2013; Lauber et al. 2008; Lladó et al. 2018; Prescott and Grayston 2013; Rousseau et al. 2024). These soil characteristics are, in turn, partially regulated by vegetation structure, which affects microclimatic conditions, organic matter inputs, and soil heterogeneity. As a result, forest structural attributes may also indirectly influence microbial communities through their effects on the soil environment (Fernandez et al. 2024; Gömöryová et al. 2013; Lang et al. 2023). Understanding these linkages is essential for assessing how structural above-ground ecosystem-scale changes impact biodiversity, nutrient cycling, and soil fertility in forest ecosystems.

Forest structure plays a fundamental role in shaping biodiversity by creating a variety of ecological niches that support diverse communities of organisms (Hilmers et al. 2018; Noss 1999; Nyberg et al. 1987). Forest structural complexity—defined by variation in tree height, canopy layering, and understory configuration—has been widely recognized as a driver of species richness and composition, both for vertebrate and invertebrate taxa (Moning & Müller 2009; Spies & Franklin 1996; Zeller et al. 2023). As forests grow towards an older stage, their increasing structural complexity, which enhances biodiversity by supporting diverse habitats and niches (Franklin and Van Pelt 2004; Hilmers et al. 2018; Lang et al. 2023). However, while the relationship between forest structure and vertebrate and invertebrate diversity has been extensively documented and well understood, its influence on belowground microbial communities remains understudied (Hamada et al. 2014; Lang et al. 2023).

Forest stand structure can influence soil properties through several mechanisms. Canopy structure regulates light penetration, temperature, and moisture availability, all of which affect microbial activity (Baldrian 2017; Gömöryová et al. 2013). The accumulation of leaf litter and woody debris contributes to soil organic matter pools, while root exudates shape microbial interactions in the rhizosphere (Birch et al. 2023; Koranda et al. 2013; Mitchell et al. 2012; Uroz et al. 2016). Moreover, tree size and stand density regulate belowground competition for water and nutrients, which in turn forms the spatial distribution of nutrients and microbial assemblages. Larger trees and denser stands tend to extract greater amounts of soil nutrients due to increased root biomass and uptake demand (Dreyling et al. 2022; Fernandez et al. 2024). At the same time, they also contribute more organic inputs to the soil through litter deposition and root exudation, enhancing microbial activity and altering community composition (Birch et al. 2023). Together, these opposing effects influence soil nutrient dynamics and microbial spatial patterns. Structural complexity further contributes to soil heterogeneity due to the presence of various cohorts of roots and various sizes of wood debris (Baldrian 2017). Soil heterogeneity is a major determinant of microbial diversity patterns. Despite these known interactions, studies explicitly linking forest structure with soil microbial communities remain scarce, and the relative contribution of different structural attributes to microbial diversity and composition remains unclear (Averill et al. 2021; Karimi et al. 2019; Lang et al. 2023).

A major limitation of previous research is the reliance on small-scale field studies that do not capture landscape-level patterns in microbial diversity (Legeay & Hijri 2023; Maron et al. 2011). Advances in remote sensing, particularly Light Detection and Ranging (LiDAR), provide an opportunity to quantify forest structure with high precision across broader spatial scales (Hamada et al. 2014; Kane et al. 2010; LaRue et al. 2019). LiDAR-derived metrics offer detailed three-dimensional representations of canopy structure that are difficult to measure using traditional field-based methods (Lefsky et al. 1999; White et al. 2016). These metrics allow the assessment of key structural attributes and complexity such as stand height, vertical layering, and canopy heterogeneity stand density, understory configurations that can influence light penetration and microclimate, indirectly affecting soil properties (Lladó et al. 2017). For example, height-related LiDAR metrics represent tree size (tall trees) which affecting soil nutrient dynamics due to a positive correlation of tree shoot (height and width) and root system size (Tumber-Dávila et al. 2022). Other metrics that related to canopy heterogeneity, canopy gaps, and vertical layering represent the complexity of forest canopy coverage which regulates the amount of sunlight and water penetration to forest ground and influence the dynamics of soil chemical properties, including soil pH and soil nutrients (Härdtle et al. 2003; McEwan et al. 2005).

When remote sensing combined with environmental DNA (eDNA) sequencing, these data can help elucidate how forest structure influences soil microbial diversity at larger spatial scales, with implications for biodiversity monitoring and forest management (Lang et al. 2023). Despite the advantages of remote sensing, field measurements remain essential for capturing fine-scale complexities of forest structure. For example, Kane et al. (2010) demonstrated that combining LiDAR with field-measured attributes yielded a strong correlation with forest structural complexity (r: 0.95). Since structural complexity indirectly influences soil microbial diversity via its effect on soil properties (Baldrian 2017; Lang et al. 2023), the integration of both remote sensing and field data is also crucial.

In this study, we investigate the effects of forest structural attributes on soil microbial diversity and community composition in mixed temperate forests. Specifically, we aim to (i) assess how stand-level attributes, structural complexity, and understory configuration predict variations in soil microbial diversity and community composition and (ii) determine the relative contribution of each attribute to explaining these variations. We hypothesize that forest structural complexity, particularly vertical layering and canopy heterogeneity, is a key predictor of microbial diversity, as it contributes to soil heterogeneity and resource availability. Our study focuses on alpha and beta diversity of soil microbial communities, as they are key indicators that offer fundamental insights into microbial community structure and variation across different forest types (Fierer & Jackson 2006; Lang et al. 2023; Tedersoo et al. 2014). These diversity measures are particularly useful for assessing broad-scale patterns of microbial diversity (George et al. 2019; Skidmore et al. 2025), providing a comprehensive perspective on community responses to environmental gradients, and do not depend on the quality and completeness of taxonomic and functional reference databases (Blackman et al. 2024; Sansupa et al. 2021).

By integrating LiDAR-derived forest structure variables with high-throughput eDNA sequencing, this study provides new insights into the role of forest structure in regulating belowground biodiversity and highlights the potential for remote sensing to enhance landscape-scale microbial monitoring.

## Material and methods

### Study site description and sampling design

**Fig. 1:**
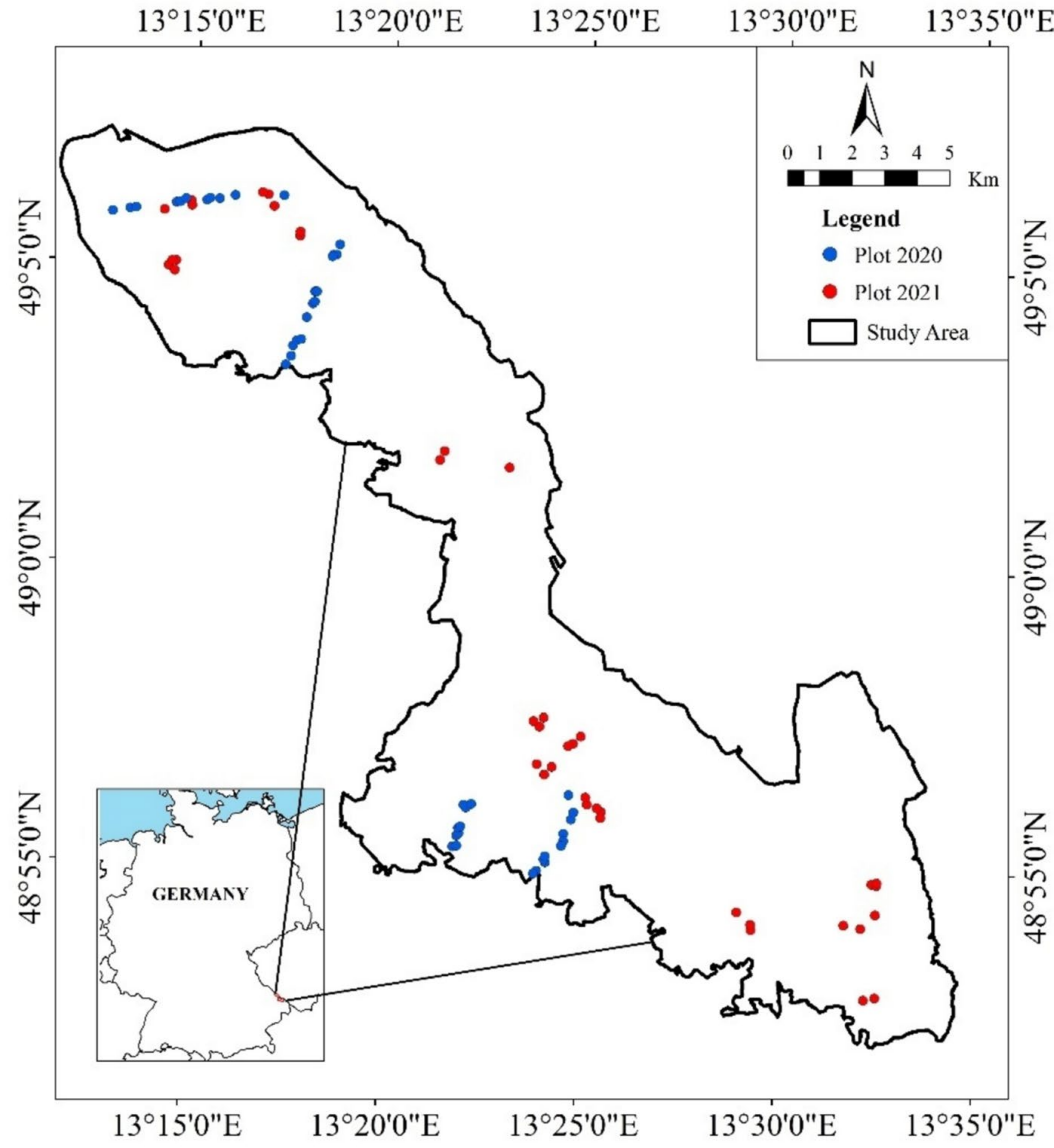
Map of field plot (30 × 30 m) distribution in Bavarian Forest National Park

Soil samples and forest structural attributes were collected within 30 m × 30 m plots in Bavarian Forest National Park (BFNP). The BFNP is the oldest national park in Germany, located in the southeastern part of the country (43.055° N, 13.203° E) (see Fig. 1). BFNP is part of the larger Bohemian Forest Ecosystem (Latifi et al. 2021), characterized by a mountainous mixed forest landscape ranging from 600 to 1453 m above sea level. The forests in BFNP are dominated by Norway spruce (Picea abies) and European beech (Fagus sylvatica). Norway spruce dominates the higher elevations (above 950 m asl), while it cooccurs with European beech and silver fir (Abies alba) at lower elevations (Cailleret et al. 2014; Heurich & Englmaier 2010). Based on the land cover map of BFNP, mixed forest areas are generally distributed across the park, and most coniferous stands (i.e., Norway Spruce) are mostly concentrated in the north side of the park (see the maps in the Supplementary S4). Established in 1970, BFNP adopted a no-intervention management strategy in some areas starting in 1983, with 75% of the park currently under this regime (Heurich et al. 2015). The park is dominated by mature and old-growth stands, including "relic forests" over 300 years old (Heurich & Englmaier 2010; Moning & Müller 2009). The soil in Bavarian Forest National Park primarily consists of oligotrophic, acidic spodo-dystric cambisol, with smaller areas of dystric histosol, derived from gneiss and granite parent material (Panagos et al. 2022; van der Knaap et al. 2020). According to Rousseau et al. (2024), the soils of our sample plots have a range of pH of 3.02 to 4.48 and a range of organic matter content (%) of 6.5 to 47.5, respectively.

The samples were collected during two field campaigns in 2020 (N = 45) and 2021 (N = 40). Both campaigns were conducted in the months July and August when deciduous trees had fully developed their foliage. All plots were distributed from 600 to 1200 masl, where most of the forested areas in BFNP are located. To maximize variation in forest structure, the sample plots were stratified over forest development stages, specifically mature and old forest stands, based on stand age information obtained from the Datapool initiative for the Bohemian Forest Ecosystem (Latifi et al. 2021), and following the stand age stratification from Oliver and Larson (1996) where stands between 80 and 150 years old are classified as mature and older stands as old growth. Old growth forest are characterized by high structural complexity, consisting of a cohort of different age classes, an abundance of deadwood, various tree heights, a multilayer canopy, and large canopy gaps (Barredo et al. 2021; Donato et al. 2012; Franklin & Van Pelt 2004; Wirth et al. 2009). Mature forests are structurally less complex than old forest stands and show more uniform structures with denser canopies, fewer gaps, and no multilayer canopy (i.e., less complex understory profile). We did not specifically sample young forest stands (less than 80 years old) since the BFNP landscape is dominated by mature and old stands. The young stands (N=20) were assigned to the mature stand class.

### Forest structure attributes generated from field measurement

In each plot, stand-level forest structural attributes—mean diameter at breast height (DBH), basal area, stand volume, and stand density—were measured (Table 1). See the detailed formula for each measurement in the supplementary document (S3). These attributes are commonly used to characterize forest structure and development stages (Burrascano et al. 2013; Ziegler 2000). Measurements were taken during the 2020 and 2021 field campaigns, focusing on a minimum of the ten largest overstory trees or approximately 30% of the dominant tree species per plot, following Gerzon et al. (2011) for forest development stage measurements.

**Table 1:** The structural attributes and Soil Properties used in this study derived from field measurements and airborne LiDAR analysis. The field-based measurement structural attributes were collected and calculated within a plot of 30 × 30 m. For the LiDAR-based structural attributes, LiDAR metrics analysis was the approach to generate two forest structural categories, i.e., structural complexity (six metrics) and understory configuration (4 metrics). A normalized gridded LiDAR image with a pixel resolution of 30 × 30 m was used to generate LiDAR metrics

Source	Category	Structural and Soil Attribute	Unit	Mean	Range
Field measurement	Stand-level Attributes	Mean Diameter at Breast Height (DBH)/Stand DBH	m	39.61	19—73
		Basal Area	m^2^/ha	1.2	0.22—2.62
		Stand Volume	m^3^/ha	49.72	12.27—93.13
		Stand Density	trees/ha	451.46	154—1120
	Soil Properties	Soil pH	Unitless	3.58	2.83—4.42
		Soil C	Percent	14.15	4.59—44.67
		Soil N	Percent	0.74	0.2—2.47
LiDAR	Structural Complexity	Maximum tree height (**zmax**)	m	36.3	21.83—49.66
		Cumulative percentage of return in 9th layer (overstory) (**zpcum9**)	Percent	98.35	87.39—99.94
		Canopy Vertical layering (Vertical Complexity Index (**VCI**))	Unitless	0.69	0.45—0.86
		Canopy surface heterogeneity (**Rumple Index**)	Unitless	4.04	0.72—7.97
		Vegetation Area Index (**VAI**)	Unitless	4.55	2.35—7.19
		Gap Fractions	Percent	0.94	0.89—0.97
	Understory Configuration	Cumulative percentage of return in 1th layer (understory) (**zpcum1**)	Percent	2.59	0.04—13.63
		Returns percentage classified as “ground” (forest floor) (**pground**)	Percent	9.38	2.49—28.05
		Percentage in 4th returns or last returns (lowest height) (**p4th**)	Percent	3.85	1.26—5.86
		Height at 10th percentile (**zq10**)	m	5.94	0—24.47

*The bold texts are the terms used throughout this study

### Generating LiDAR metrics as a forest structural representation

LiDAR data were acquired using an airborne laser scanner (ALS) equipped with a Riegl LMS-Q680i LiDAR sensor (1550 nm wavelength, 0.5 mrad beam divergence). The data were collected over the entire BFNP in June 2017, under leaf-on conditions, with a flight altitude of 550 m and a point cloud density ranging from 30 to 70 points/m^2^ (Zong et al. 2021). The point cloud was normalized using a cloth simulation function (Zhang et al. 2016) and gridded to 30 m resolution according to the dimension of the field plot. The gridded image was then transformed into LiDAR metrics using point cloud statistics such as maximum and minimum values, standard deviation, coefficient of variation (CV), skewness, kurtosis, and mean within each 30 x 30 m forest sample plot.

**Fig. 2:**
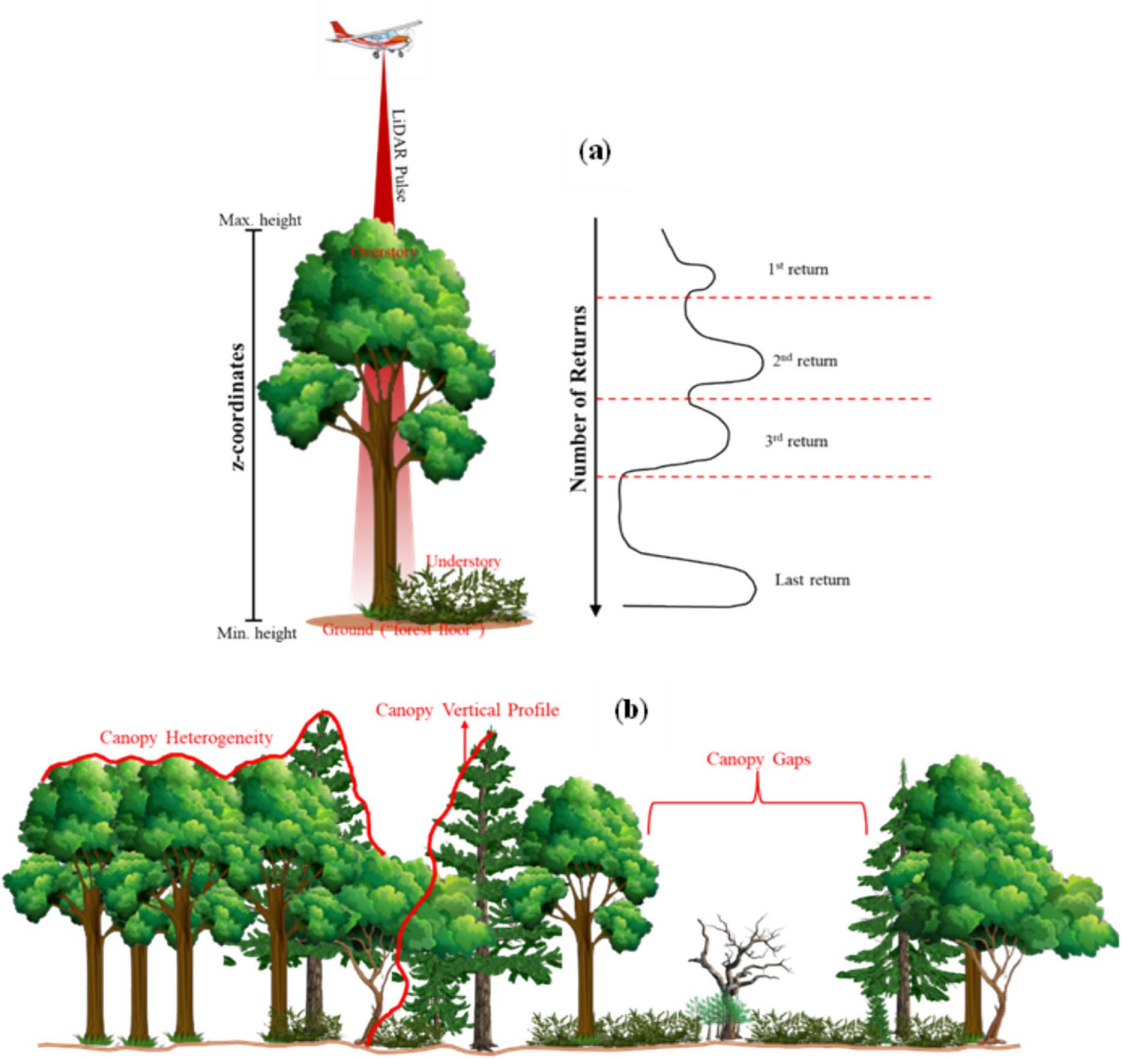
An Illustration of the concept of LiDAR pulse characteristics used to derive LiDAR metrics in this study. (a) depicts the characteristics of z-coordinates and number of returns, and (b) depicts the advanced LiDAR metrics that capture the canopy horizontal (heterogeneity) and vertical profile. Later, the derived metrics were categorized into structural complexity and understory configuration categories. See Table 1 for information on the LiDAR metrics used in this study

We divided the LiDAR metrics into two groups, i.e., Structural Complexity and Understory Configuration. Each group contains metrics that are derived based on the LiDAR pulse characteristics, i.e., z-coordinate and number of returns. The z-coordinate represents the elevation of a point within a point cloud relative to a reference surface. The number of returns is the number of emitted pulses that return to the sensor after interacting with the objects. The first return represents the first object the laser hits, such as a tree canopy, and the last return represents the final reflection, typically from the ground or the lowest detected surface. Additionally, some metrics were derived from the canopy height model (CHM) that cover vertical and horizontal (heterogeneity) profiles (Ayrey et al. 2019; Bakx et al. 2019). CHM was generated from the normalized point clouds and interpolated into a 1 m resolution raster cell using Delaunay triangulation (Roussel et al. 2020). CHM-derived metrics are the vertical complexity index (VCI) (van Ewijk et al. 2011), canopy surface heterogeneity (Rumple Index) (Kane et al. 2010), gap fractions (Atkins et al. 2018), and vegetation area index (VAI) (Bouvier et al. 2015). See the illustration of the LiDAR pulse characteristics concept in Figure 2. See the illustration of the LiDAR pulse characteristics concept in Figure 2. To obtain metrics for each plot, we clipped an area of 3 by 3 pixels in the LiDAR metrics dataset with the plot’s coordinate as the center. This is to ensure that extracted metrics fall within our measurement plot.

Raw lidar point cloud data were imported in the R environment using the *lidR* package (Roussel et al. 2020), which was used to calculate LiDAR metrics. A total of 10 metrics were selected, which represent relevant features of forest structure (Adiningrat et al. 2025) and showed low levels of multicollinearity (variance inflation factor ≤ 5; Hair et al. 2018). See the detailed description and formula of each metric in Supplementary Table 1.

### Soil eDNA sampling procedure

Three (2020) or two (2021) representative composite bulk soil samples were collected per plot (30 m × 30 m) using a 5 cm diameter x 10 cm depth corer (Siegenthaler et al. 2024). Each composite sample consisted of nine subsamples from the top 10 cm of soil (after litter removal), collected in 3 × 3 m grid subplots. The subplots were randomly distributed within the 30 m × 30 m plots. Collection of eDNA data in multiple subplots per plot allowed for a better representation of the soil communities, which typically show high spatial heterogeneity (Averill et al. 2021), while spatially aligning the eDNA data with the Stand-level Attributes (e.g., basal area, stand volume) collected at the 30 × 30 m spatial plot level. The center of each 30 x 30 m plot was georeferenced using a Differential Global Positioning System (DGPS) Leica GPS 1200 (Leica Geosystems AG, Heerbrugg, Switzerland) with an accuracy of better than 1 m after post-processing.

Each composite sample was homogenized manually, and we removed large rocks and roots. Composite samples were transported on ice and stored at ‒30° C (Rousseau et al. 2024). The composite soil samples were used as the source for eDNA extraction, soil pH measurements, and nutrient analyses. To prevent DNA cross-contamination, soil corers were sterilized with 10% bleach and rinsed with deionized water between samples. Negative field controls were obtained by collecting subsamples of the rinse water used for sterilization.

Soil pH, total N, and total C (Table 1) were analyzed following Rousseau et al. (2024). Soil properties were selected based on known associations with soil microbial diversity (Fierer & Jackson 2006; S. Jiang et al. 2021; Kopáček et al. 2013; Lauber et al. 2008; Lladó et al. 2018; Prescott & Grayston 2013; Rousseau et al. 2024) and were only used as complementary information since a detailed assessment of their relation to soil biodiversity was outside the scope of this study. Soil Properties were averaged per plot.

### Soil eDNA profile extraction and bioinformatic analyses

Soil DNA extraction and amplification protocols are described in Siegenthaler *et al*. (2023). In summary, DNA extraction (15 g of well-homogenized soil) was conducted following the saturated phosphate buffer method of Taberlet et al. (2012). The Bacterial (16S rRNA gene) and fungal (ITS rRNA region) DNA were amplified using the 515F/806R (Apprill et al. 2015; Parada et al. 2016) and ITS86/ITS4-ngs (Tedersoo et al. 2014; Turenne et al. 1999) primer sets. Field and extraction controls were combined per control type prior to amplification. Genome Quebec (Montreal, Canada) performed library preparation and Next Generation paired-end sequencing on one lane of the Illumina NovaSeq 6000 SP platform using the PE250 kit. Primers included CS1 (forward) or CS2 (reverse) adaptor sequences for multiplexing with the Fluidigm Access Array System, and an indexing PCR added indexes and i5/i7 Illumina adapter sequences to the amplicons.

Bioinformatic analyses were performed using the QIIME 2™ software suite (Bolyen et al. 2019) using the SILVA (Quast et al. 2013) and UNITE (Nilsson et al. 2019) databases for taxonomical assignments. DADA2 was used for exact Amplicon Sequence Variant (ASV) inference (Callahan et al. 2016), and LULU for post-clustering curation (Frøslev et al. 2017). An Amplicon Sequence Variant (ASV) is a highly accurate DNA sequence obtained from microbial sequencing data, representing real biological variants without sequencing errors. They provide a more accurate representation of diversity compared to clustered sequences (Callahan et al. 2017). ASV tables were further filtered for potential contaminants: taxa with maximum reads controls > maximum reads samples, non-bacterial and fungal ASVs, tag-switching (Taberlet et al. 2018) and low frequency noise (ASVs < 10 reads; (Polling et al. 2022)). See Supplementary Tables 2 A and B for details. Filtered reads were rarefied (average of 100 iterations; (Cordier et al. 2019)) to 150000 reads for 16S and 77000 for ITS, based on the rounded lowest read depth, using the *Vegan* v. 2.6-2 R package (Oksanen et al. 2022).

### Statistical analysis

All statistical analyses were performed using R v.4.2.3. DNA profiles were averaged at the plot level to align with the corresponding forest structure data. Alpha diversity indices, including observed ASV richness and Shannon diversity, were calculated using the *Vegan* v. 2.6-2 R package. Fungal communities were analyzed as a whole rather than subdivided into functional or taxonomic groups, in order to maintain analytical consistency with bacterial data and to ensure a concise and focused interpretation of structural effects on microbial diversity.

A machine learning approach using boosted regression trees (BRT) was implemented via the *caret* v. 6.0-94 package to evaluate the influence of forest structural attributes on soil microbial diversity. A 10-fold cross-validation was applied for internal validation, and the optimal model parameters (tree number and interaction depth) were tuned using the *expand.gri*d function. Model performance was assessed using R-squared and root-mean-square error (RMSE). The relationship between structural attributes and soil microbial alpha diversity was further explored using partial dependence (PD) plots, generated with the *pdp* package. A total of 14 structural attributes (four field-measured attributes and ten LiDAR-derived metrics) were used as predictors (Table 1).

Community composition analyses were conducted using permutational multivariate ANOVA (PERMANOVA) with 999 permutations. Bray–Curtis dissimilarities, derived from Hellinger-transformed ASV counts, were used to quantify differences in microbial community composition. PERMANOVA was applied to test the effects of forest structural attributes on microbial community composition using the adonis function in the *Vegan* v. 2.6-2 package.

## Results

### Effect of forest structures on soil microbial diversity

The boosted regression tree (BRT) model used fourteen forest structural attributes to predict bacterial and fungal ASV richness and Shannon diversity indices. For ASV richness, the model explained more variation in fungal diversity (R^2^ = 0.74, RMSE = 45.47) than in bacterial diversity (R^2^ = 0.57, RMSE = 968.07) (Table 2). Similarly, fungal Shannon diversity was better predicted by forest structural attributes (R^2^ = 0.51, RMSE = 0.41) than bacterial Shannon diversity (R^2^ = 0.43, RMSE = 0.36).

**Table 2:** Proportion of explained variance (R2) and Root Mean Squared Error (RMSE) of the Boosted Regression Tree model fit of soil alpha diversity with complex forests-associated structural attributes and soil properties

Diversity	Bacterial		Fungal	
	R^2^	RMSE	R^2^	RMSE
Richness	0.57	968.07	0.74	45.47
Shannon Index	0.43	0.36	0.51	0.41

**Fig. 3:**
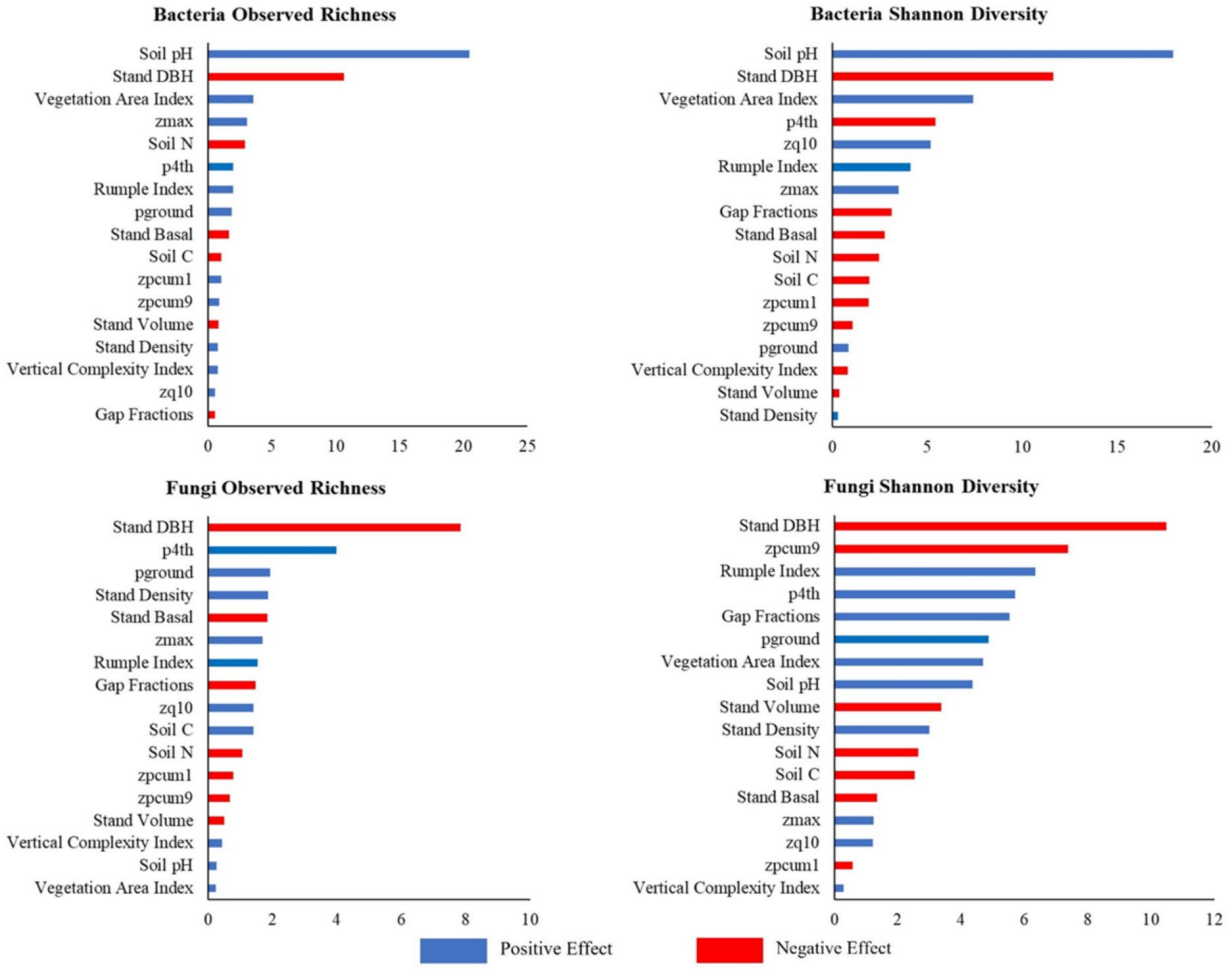
Variable importance (VI) and effect of forest structural attributes and soil properties in predicting soil alpha diversity using boosted regression trees. The importance rank was generated from boosted regression trees, while the effects were analyzed using a partial dependence approach

Overall, forest structural attributes were stronger predictors of ASV richness than Shannon diversity for both bacterial and fungal communities, as indicated by higher R^2^ values for richness across both microbial groups. The BRT analysis also highlighted the key predictors for each diversity index (Fig. 3), with DBH consistently emerging as the most important predictor for both bacterial and fungal diversity. Notably, stand DBH exhibited a negative relationship with the alpha diversity of both microbial groups, suggesting that larger trees may correspond to lower microbial diversity in the soil. Furthermore, the variable importance showed that soil pH was the most important variable, as expected, especially for bacteria. However, other soil properties (i.e., soil N and soil C) were not as important as forest structural attributes, demonstrated by their lower rank than several structural attributes (Fig. 3).

Overall, stand-level attributes such as DBH, basal area, and volume were negatively associated with microbial alpha diversity, indicating reduced bacterial and fungal richness in plots dominated by larger trees. In contrast, structural complexity metrics—such as canopy height, Rumple Index, and VCI—exhibited positive associations, suggesting that more complex forest structures support greater microbial diversity. Understory configuration also showed generally positive, though more variable, relationships.

### Effect of complex forest structures on soil microbial community composition

PERMANOVA analyses revealed that several forest structural attributes significantly influenced bacterial and fungal community composition. However, the overall variation explained by these attributes was relatively modest, with total R^2^ values below 0.27. Individual structural attributes explained between 1% and 3% of the variation in microbial community composition (Table 3).

**Table 3:** Effects of forest structural attributes and soil properties on the soil bacterial and fungal community composition, tested by PERMANOVA with Bray–Curtis dissimilarities constructed from Hellinger-transformed read counts

BACTERIA						
Predictor	**Df**	**Sum Of Sqs**	**R**^**2**^	**F**	**Pr(> F)**	
Rumple Index	1	0.24	0.02	1.62	0.03	*
VCI	1	0.18	0.01	1.19	0.18	
GapsFr	1	0.17	0.01	1.14	0.24	
zmax	1	0.17	0.01	1.11	0.25	
VAI	1	0.19	0.01	1.27	0.12	
zq10	1	0.14	0.01	0.90	0.58	
zpcum1	1	0.19	0.01	1.26	0.14	
zpcum9	1	0.16	0.01	1.07	0.29	
p4th	1	0.19	0.01	1.29	0.11	
pground	1	0.17	0.01	1.14	0.25	
DBH_mean	1	0.39	0.02	2.61	0.00	**
Density	1	0.17	0.01	1.12	0.24	
Basal	1	0.23	0.01	1.51	0.04	*
Volume	1	0.23	0.01	1.50	0.06	
soil_pH	1	1.10	0.07	7.31	0.00	***
TC	1	0.28	0.02	1.86	0.02	
TN	1	0.18	0.01	1.22	0.16	
R^2^			**0.27**			
Residual	**66**	**9.8917**	**0.73**			
FUNGI						
Predictor	**Df**	**Sum Of Sqs**	**R**^**2**^	**F**	**Pr(> F)**	
Rumple Index	1	0.45	0.02	1.48	0.02	*
VCI	1	0.39	0.01	1.28	0.07	
GapsFr	1	0.37	0.01	1.21	0.11	
zmax	1	0.31	0.01	1.04	0.32	
VAI	1	0.35	0.01	1.16	0.15	
zq10	1	0.34	0.01	1.14	0.19	
zpcum1	1	0.32	0.01	1.07	0.29	
zpcum9	1	0.38	0.01	1.24	0.10	
p4th	1	0.37	0.01	1.22	0.10	
pground	1	0.30	0.01	1.00	0.45	
DBH_mean	1	0.65	0.02	2.14	0.00	***
Density	1	0.32	0.01	1.07	0.30	
Basal	1	0.37	0.01	1.23	0.11	
Volume	1	0.42	0.01	1.40	0.04	*
soil_pH	1	0.76	0.03	2.51	0.00	***
TC	1	0.35	0.01	1.17	0.18	
TN	1	0.29	0.01	0.95	0.53	
R^2^			**0.23**			
Residual	**66**	**19.8748**	**0.77**			

*** *p* < 0.001; ** *p* < 0.01, * *p* < 0.05

Among the structural attributes, stand DBH and Vegetation Area Index (VAI) were significant predictors of both bacterial and fungal community composition (p < 0.05). Maximum tree height (zmax) significantly explained variation in bacterial community composition (p < 0.05), but not fungal composition. In contrast, structural diversity metrics, including canopy heterogeneity (Rumple Index) and vertical complexity index (VCI), alongside stand volume, significantly explained variation in fungal community composition (p < 0.05). These findings suggest that while forest structural attributes are important in shaping soil microbial communities, their effects differ between bacterial and fungal groups, with structural complexity playing a more prominent role in influencing fungal communities.

## Discussion

A set of structural attributes, which comprises stand-level attributes, structural complexity, and understory configurations, contributes significantly to explaining the variation in microbial diversity and community composition in the mixed temperate forest in BFNP. Our results demonstrated that the arrangement of forest structural attributes influences the diversity pattern of soil microbial communities (i.e., bacterial and fungal). Furthermore, our results confirm a study from Lang et al. (2023), where forest structures can moderately explain soil microbial diversity at a soil depth of 10 cm. Overall, structural attributes are more important for predicting soil microbial richness than Shannon diversity for both bacterial and fungal communities. This is possibly connected to difficulties in accurately assessing soil microbial relative abundances (Carini et al. 2016) since Shannon diversity incorporates both species richness and evenness.

When comparing bacterial and fungal diversity, we found that forest structural attributes were better predictors for fungal diversity than for bacterial diversity, as indicated by higher R^2^ values for both ASV richness and Shannon diversity. This suggests that fungal diversity is more closely linked to forest structure, particularly tree traits. Soil fungi tend to be more strongly associated with forest structural attributes than bacteria, possibly due to their capacity to carry plant traits into the bulk soil extending from the rhizosphere, especially the root-associated fungi (Urbanová et al. 2015). However, this relationship may vary across fungal functional guilds (i.e., symbiotrophs, saprotrophs, and pathotrophs), each of which may respond differently to forest structural variation (Nguyen et al. 2016). Host tree traits can directly influence soil fungal communities, especially symbiotrophic fungi (i.e. ectomycorrhizal taxa) and pathotrophs, via tree root traits, nutrient exchange dynamics, root exudates, or host defense compounds with antimicrobial properties (Odriozola et al. 2020; van der Heijden et al. 2008). Forest structural complexity, including variations in stand height, canopy openness, and tree density, may also exert indirect effects on fungal and bacterial communities by regulating the soil microclimate (e.g., temperature and moisture regimes) (Fernandez et al. 2024; Odriozola et al. 2020). These structural attributes may also regulate belowground carbon inputs through root biomass and turnover, further shaping soil microbial community assembly (Baldrian 2017).While bacteria also interact with plants and trees, their associations are generally less specialized and weaker compared to fungi, particularly with respect to plant functional diversity (Hanif et al. 2019).

The selected soil properties in our study demonstrated an expected pattern of effects, especially reduced diversity at lower soil pH (Fierer & Jackson 2006; Lang et al. 2023; Rousseau et al. 2024). We found that incorporating soil properties, especially soil pH, improved our predictive models (Figure 3, Table 3). However, other soil properties (N, C) did not alter our conclusion regarding the influence of forest structural attributes, and their importance is less than that of structural attributes in the model predictions.

In terms of microbial community composition, structural complexity, along with stand DBH and maximum tree height (zmax), explained 23–27% of the variation at the landscape level. The unexplained variance in microbial community composition is consistent with other landscape-level studies on soil microbiomes and may be attributed to the highly heterogeneous nature of microbial communities at small spatial scales (Karimi et al. 2019; Nunan et al. 2002; O’Brien et al. 2016). Additionally, the disparity between the spatial scales at which microbial communities are characterized and the canopy structural attributes measured may reduce the accuracy of predicting microbial community composition, as above- and belowground components become increasingly decoupled at larger spatial scales (Lang et al. 2023; Martiny et al. 2011).

Forest structural attributes showed inconsistent effects on soil microbial diversity, depending on the specific features considered. Variables representing structural complexity (such as Vegetation Area Index, Rumple Index, and p4th) were generally positively associated with microbial diversity. At the same time, tree size and stand-level characteristics (including stand DBH, volume, basal area, and tree density) showed negative relationships with microbial diversity. These contrasting effects of forest structures on soil microbes depict the inconsistent relationship between forest stage development and soil microbial diversity, as reported by several studies, especially in mature or older forest stages (Gömöryová et al. 2013; Odriozola et al. 2020; Ren et al. 2019; Zeng et al. 2022). Soil microbial diversity generally increases from mature to old-growth forest stages, driven by increasingly complex forest structures that support a greater variety of microhabitats, canopy stratification, and vegetation composition (Gömöryová et al. 2013; Spînu et al. 2022). At the same time, the positive effect of tree size on soil microbial diversity seems to decrease in older forests as trees reach their growth and developmental peaks (Zeng et al. 2022). Another factor that contributes to the slight decline of soil microbial diversity in older forest stages can be caused by the increased environmental filtering in these stable environments (Dreyling et al. 2022; Odriozola et al. 2020; Zeng et al. 2022). In this case, increased tree size and stand-level characteristics can be considered an environmental filter for soil microbial diversity, representing more stable and established forest environments. By evaluating the contrasting effects of structural complexity, tree size, and stand-level characteristics, this study advances our understanding of how forest developmental stages influence soil microbial diversity.

A notable finding from our study is the consistent role of tree size, represented by stand DBH, as the most important structural variable influencing soil microbial diversity (Fig. 3, Table 3). Stand DBH being consistently prominent compared to the other structural attributes may also be related to its characteristic that reflects individual tree size and biomass allocation, which directly affects root exudation, litterfall, and microhabitat formation around tree bases, creating localized hotspots of microbial activity (Prescott & Grayston 2013). In contrast, other structural attributes—such as stand density, basal area, or structural complexity indices (e.g., Rumple Index, VAI)—may not capture fine-scale spatial variation. These metrics are typically aggregated rather than based on individual-level measurements, making them more suitable for describing broader spatial patterns, such as spatial arrangement and ecological niche space (Franklin & Van Pelt 2004; LaRue et al. 2019). Furthermore, Stand DBH was negatively associated with microbial diversity, indicating that microbial diversity tends to decline in stands dominated by large trees. This may be related to the immobilization of soil nutrients in biomass as trees grow larger, reducing the availability of nutrients in the soil and potentially limiting microbial diversity (Binkley et al. 1995; Dreyling et al. 2022; Ryan et al. 1997). Stands with large DBH values also support extensive root systems, which can create a larger rhizosphere (the soil zone surrounding roots). The rhizosphere tends to have higher microbial densities but lower diversity compared to bulk soil due to root exudation and rhizodeposition (Zhou et al. 2022). In contrast, stand height attributes generally had a positive effect on soil microbial diversity, likely because greater variation in tree heights creates more diverse microhabitats within the soil, fostering a wider range of microbial niches (Spînu et al. 2022).

In addition to tree size, structural complexity—represented by attributes such as canopy vertical layering, vegetation area index, and canopy heterogeneity (Rumple Index)—was a significant driver of variation in soil microbial community composition, supporting the findings of Lang et al. (2023). Canopy layering and vegetation area index capture the diversity and complexity of vertical canopy profiles, which can indirectly influence soil chemical properties (e.g., pH, nutrient availability) by regulating light and water penetration to the forest floor (Härdtle et al. 2003; McEwan et al. 2005). As forests mature, the increasing heterogeneity of the soil environment may create new niches, allowing microbial diversity and composition to diversify (Gömöryová et al. 2013). However, this diversity appears to reach an asymptote at the climax stage of forest development, as our study found.

Interestingly, canopy heterogeneity had a significant effect on fungal community composition but not on bacterial composition. Fungi, which play an essential role as decomposers, are closely linked to the availability of organic matter controlled by the complexity of the canopy's horizontal structure (Gömöryová et al. 2013; Lang et al. 2023). Canopy heterogeneity arises from variations in tree diameters, heights, gaps, and deadwood, as well as the presence of pioneer species (Franklin & Van Pelt 2004). These factors influence tree productivity and biomass, affecting the rate of organic matter deposition on the forest floor, thus benefiting fungal communities (Nguyen et al. 2016).

Unexpectedly, structural attributes related to understory configuration were important for predicting soil microbial diversity but did not significantly explain variation in community composition. This may suggest that community composition is more influenced by the diversity of understory vegetation species than by structural configuration alone (Eisenhauer et al. 2011; Gömöryová et al. 2013). Diverse understory species, such as shrubs and herbaceous plants, can diversify the range of substrate sources, directly and indirectly affecting microbial relative abundance and community composition (Chen et al. 2021). The proximity of understory vegetation roots to microbial communities, especially those of shrubs and herbaceous plants, could further enhance this effect (Chen et al. 2021).

Our results suggest that airborne LiDAR-derived structural attributes can effectively estimate and predict microbial diversity at landscape scales. As airborne LiDAR and other remote sensing technologies can cover large spatial extents, they offer promising tools for scaling up predictions of soil microbial diversity across broader areas. Additionally, some field-measured attributes in our study could be substituted or modeled using LiDAR metrics, as field and LiDAR-derived attributes are often correlated (Kane et al. 2010). Integrating LiDARderived forest structural attributes with soil variables, such as the soil buffer capacity information, could also be an important consideration for future studies to reliably assess the strength of the stand effect. Soil buffering capacity refers to the ability of soil to absorb or release compounds that affect acidity or alkalinity while maintaining a relatively stable pH, and is typically associated with organic matter, texture, and the presence of certain minerals (Nelson and Su 2010). For example, the soil acid buffering capacity in mature forests is higher than in young forests due to the higher concentrations of soil organic matter and Al and Fe in the soil (Jiang et al. 2016). Additionally, the presence of certain minerals that influence soil microbial activity and soil health is related to forests with higher stand density, larger canopy volume (characterized by big trees), and multiple vertical layers (Židó et al. 2022).

In this study, we focus on alpha and beta diversity as robust and widely used indicators of microbial community structure and spatial variation, offering reliable insights across forest types without relying on potentially incomplete taxonomic or functional reference databases. Future studies should explore higher taxonomic levels and incorporate additional covariates that capture ecological interactions and environmental filters, allowing for a more nuanced understanding of diversity changes at different spatial and temporal scales (Averill et al. 2021). Expanding the analysis to include functional or compositional traits could also be the focus of future studies. Nevertheless, careful consideration is needed due to challenges related to the taxonomical and functional assignment of microbial ASVs in soil samples (Sansupa et al. 2021).

As our study focused on a mixed temperate forest in Central Europe, further research is needed to examine the relationship between forest structural attributes and soil microbial diversity in other forest types and biomes. Additionally, the diversity and composition of ground vegetation species, which play a critical role in influencing soil microbial diversity, should be incorporated into future studies (Chen et al. 2021; Lang et al. 2023). Close-range sensing technologies such as terrestrial laser scanners or UAV-based LiDAR may be more effective for identifying ground vegetation diversity, as airborne LiDAR is limited by resolution and canopy occlusion (Lang et al. 2023).

## Conclusions

In line with the EU 2030 Soil Strategy, understanding soil microbial diversity is critical for informing soil protection and management strategies in response to land-use changes and overexploitation in the agricultural and forestry sectors (European Commission, 2021). This study demonstrates that variations in soil microbial diversity and community composition within forest ecosystems can be partially predicted and explained using a set of forest structural attributes. Specifically, soil microbial richness and diversity can be effectively modeled based on forest stand-level attributes, height structure, understory configurations, and structural complexity. Community composition, while more complex, is also primarily influenced by these forest structural attributes.

Unlike macrofauna diversity, which generally increases with greater structural complexity in older forests, soil microbial diversity appears to remain stable or increase only slightly as forest structural complexity increases. This suggests that different ecological processes govern the diversity of soil microbes and macrofauna. Nevertheless, forest structural attributes serve as useful proxies for upscaling the assessment of soil microbial diversity to larger spatial scales, such as the landscape level.

Remote sensing technologies, particularly LiDAR, provide a valuable tool for assessing forest structure over large areas. In this study, we demonstrated that LiDAR-derived structural attributes—such as structural complexity and understory configuration—can be used to predict soil microbial diversity. This highlights the potential of remote sensing for monitoring belowground biodiversity at broader spatial extents, such as the landscape level, offering critical insights for conservation and protected area management.

## Supplementary Information

Below is the link to the electronic supplementary material.

ESM1 (DOCX. 3.91 MB)

## Data Availability

The datasets generated during this study as well as the source datasets are available upon request to the corresponding author.
