# Supplementary material for: Effect of forest structural attributes on soil microbial diversity in mixed temperate forests
Source: Plant Soil. 2025 Oct 1;517(1):867–85. doi: 10.1007/s11104-025-07907-4 (PMC12789230; doi:10.1007/s11104-025-07907-4)
Supplement: Supplementary file 1 — (DOCX. 3.91 MB) [file 11104_2025_7907_MOESM1_ESM.docx]

**SUPPLEMENTARY MATERIAL**

**Effect of structural attributes on soil microbial diversity**

**in structurally complex mixed temperate forests**

*Devara P. Adiningrat ^a^*, Andjin Siegenthaler ^a^, Michael Schlund ^a^, Tiejun Wang ^a^, Andrew K. Skidmore ^a,^ Mélody Rousseau ^a^, Marco Heurich ^b,c,d^*

*a Faculty of Geo-Information Science and Earth Observation, University of Twente, Enschede, The Netherlands.*

*b Department of National Park Monitoring and Animal Management, Bavarian Forest National Park, Freyunger Str. 2, 94481 Grafenau, Germany.*

*c Faculty of Environment and Natural Resources, University of Freiburg, Tennenbacher Str. 4, 79106 Freiburg, Germany.*

*d Department of Forestry and Wildlife Management, Inland Norway University of Applied Sciences, Evenstads Vei 80, 2480 Koppang, Norway.*

*Corresponding author: Email address: d.p.adiningrat@utwente.nl

**Supplementary Table S1**. The description and formula of each Airborne LiDAR metric which was used in this study.

| **Metrics** | **Description** | **Formula** |
| --- | --- | --- |
| zmax | Maximum object’s height | *z_max_* = max ($z_{i}$)  where z_i_​ is the height of the ith return. |
| zq10 | Decile (q=10) of height values. Equivalent to the 10th percentile | *z_q10_* = Quantile ($z_{i}$, 0.10)  similar to zpcum1, but the **calculation method might differ slightly** (e.g., interpolated vs. non-interpolated quantile). zq10 is usually from the quantile_z() function |
| zpcum9 | Cumulative percentage of return in 9^th^ layer (overstory) | *z_pcum9_* = Quantile ($z_{i}$, 0.90)  where the quantile is calculated based on **cumulative point count** |
| zpcum1 | Cumulative percentage of return in 1^th^ layer (understory) | *z_pcum1_* = Quantile ($z_{i}$, 0.10)  where the quantile is calculated based on **cumulative point count** |
| p4th | Percentage in 4^th^ returns or last returns (lowest height) | $p_{4th}=\frac{\sum_{i=1}^{n} \parallel(z_{i}>4)}{n}$  where ∥ is the indicator function (1 if condition true, 0 otherwise), and nnn is the number of returns. |
| pground | Returns percentage classified as “ground” (forest floor) | $p_{ground}=\frac{\sum_{i=1}^{n} \parallel({class}_{i}=ground)}{n}$  where class*_i_* is the classification code of the return (usually class 2 = ground in LAS/LAZ format) |
| Rumple  (Hardiman et al., 2011; Kane et al., 2010) | Ratio between outer canopy surface area and projected ground surface | Rumple Index = $\frac{A_{canopy}}{A_{ground}}$   - *A*_canopy_​ = Total surface area of the canopy derived from a mesh/triangulation of the canopy (CHM) - *A*_ground_​ = Projected ground area (e.g., area of the grid cell or plot) |
| Gap Fraction  (Atkins et al., 2018) | The proportion of LiDAR pulses that **pass through the canopy** without hitting vegetation at a given height. | Gap Fraction = $\frac{N_{below z}}{N_{total}}$   - *N*_below z_​ = number of LiDAR pulses that **do not hit vegetation above height z** (i.e., hit ground or nothing) - *N*_total​_ = total number of first returns or pulses |
| Vegetation Area Index (VAI)  (Bouvier et al., 2015; Kamoske et al., 2019) | An analog to LAI (Leaf Area Index), but derived from **discrete return LiDAR**, estimating total vegetation surface area per ground area. | VAI = $\frac{-ln(P_{gap)}}{k}$   - *P*_gap_​ = gap fraction (see below) - *k* = extinction coefficient (depends on canopy structure and LiDAR scan angle, often approximated as 0.5–1.0) |
| Vertical Complexity Index (VCI)  (van Ewijk et al., 2011) | The distribution evenness of point cloud within a vertical layer, often using Shannon entropy. | VCI = $-\sum_{i=1}^{n} p_{i}log(p_{i})$   - The canopy is divided into *n* vertical bins (e.g., 1-m height layers). - *p*_i_ = number of points in bin _i_ divided by total number of points |

**Supplementary Tables 2A and 2B**. Bioinformatic pipelines for 16S (A) and ITS (B) metabarcoding data. Steps 2-5 were conducted in QIIME 2™ and steps 5-13 in R version 4.2.3. Non-default parameters are provided.

| **Step** | **Package** | **Parameters** | **Reads** | **ASVs** | **Samples** |
| --- | --- | --- | --- | --- | --- |
| Demultiplexing | Performed by Genome Quebec |  | 2020: 173,874,448 ^1^  2021: 108,780,402 ^1^ |  | 2020: 493  2021: 338 |
| Primer trimming | cutadapt | minimum-length: 200 | 2020: 168,445,114 ^1^  2021: 103,704,032 ^1^ |  | 2020: 493  2021: 338 |
| Denoising and ASV merging | dada2 | trunc-len-f: 232  trunc-len-r: 230  MaxEE: 2 | 2020: 116,120,466  2021: 69,349,773 | 2020: 149,143  2021: 106,659 | 2020: 493  2021: 338 |
| Merging of sequencing runs | QIIME 2 | Merging 2020 and 2021 Feature tables and fasta files | 185,470,239 | 222,991 | 831 |
| Sample selection | QIIME 2 | Exclusion of non-soil samples | 134,578,361 | 199,673 | 586 |
| Post-clustering curation | LULU | minimum_match: 90% ^2^  minimum_relative_cooccurence: 0.95 | 134,578,361 | 168558 | 586 |
| Blank correction | Custom R-script | Removal criteria: max reads in blanks ≥ max reads in samples | 130,679,296 | 168,023 | 571 ^3^ |
| Non-bacterial read filter | Custom R-script | Removal of reads with domain ≠ bacteria | 127,070,276 | 164,972 | 571 |
| Tag-switching | Custom R-script | Threshold: 0.003%.  Based on the percentage of positive control reads detected in the samples | 126,876,606 | 164,972 | 571 |
| Sample selection | Custom R-script | Exclusion of samples that were not pertinent to this study | 53,195,819 | 90,688 | 214 |
| Low frequency noise filtering | Custom R-script | Removal of ASVs with < 10 reads in total | 53,051,398 | 54,990 | 214 |
| Rarefaction | Vegan | Subsample size: 150,000 reads | 32,100,515 | 54,990 | 214 |
| Data pooling | Custom R-script | Averaging of 2-3 samples per plot | 12,750,347 | 54,990 | 85 |

Supplementary table 2B. Fungal (ITS) Bioinformatic pipeline.

| **Step** | **Package** | **Parameters** | **Reads** | **ASVs** | **Samples** |
| --- | --- | --- | --- | --- | --- |
| Demultiplexing | Performed by Genome Quebec |  | 2020: 155,981,020 ^1^  2021: 115,535,393 ^1^ |  | 2020: 481  2021: 337 |
| Primer trimming | cutadapt | minimum-length: 150 | 2020: 153,450,121 ^1^  2021: 112,188,405 ^1^ |  | 2020: 481  2021: 337 |
| Denoising and ASV merging | dada2 | trunc-len-f: 0  trunc-len-r: 0  MaxEE: 2 | 2020: 124,873,573  2021: 89,246,187 | 2020: 36,310  2021: 16,945 | 2020: 481  2021: 337 |
| Merging of sequencing runs | QIIME 2 | Merging 2020 and 2021 Feature tables and fasta files | 198,187,306 | 49,095 | 818 |
| Sample selection | QIIME 2 | Exclusion of non-soil samples | 133,380,401 | 31,501 | 586 |
| Post-clustering curation | LULU | minimum_match: 84% ^5^  minimum_relative_cooccurence: 0.95 | 133,380,401 | 13,044 | 586 |
| Blank correction | Custom R-script | Removal criteria: max reads in blanks ≥ max reads in samples | 132,277,375 | 12,985 | 571 ^3^ |
| Non-fungal read filter | Custom R-script | Removal of reads with domain ≠ bacteria | 130,973,249 | 9,240 | 571 |
| Tag-switching | Custom R-script | Threshold: 0.00156%.  Based on the percentage of positive control reads detected in the samples | 130,944,354 | 9,240 | 571 |
| Sample selection | Custom R-script | Exclusion of samples that were not pertinent to this study | 50,205,746 | 4,698 | 214 |
| Low frequency noise filtering | Custom R-script | Removal of ASVs with < 10 reads in total | 50,202,276 | 3,767 | 214 |
| Rarefaction | Vegan | Subsample size: 77,000 reads | 16,247,050 | 3,765 | 211 ^6^ |
| Data pooling | Custom R-script | Averaging of 2-3 samples per plot | 6,544,943 | 3,765 | 85 |

Notes:

^1^ Number of forward and reverse reads, prior to merging pair-end reads

^2^ Brandt, M. I. et al. Bioinformatic pipelines combining denoising and clustering tools allow for more comprehensive prokaryotic and eukaryotic metabarcoding. *Mol Ecol Resour* 21, 1904-1921, doi:10.1111/1755-0998.13398 (2021).

^3^ Removal of blank samples (N=9) and positive control samples (N=6)

^5^ Frøslev, T. G. *et al.* Algorithm for post-clustering curation of DNA amplicon data yields reliable biodiversity estimates. *Nature Communications* **8**, 1188, doi:10.1038/s41467-017-01312-x (2017).

^6^ Three samples contained insufficient reads for rarefaction.

**Supplementary S3**. Formula of stand-level attributes from field measurements

Mean diameter at breast height (DBH)

̅*DBH* *= (Σ dbh)*/*N*

where: ̅DBH= mean or average DBH (stand DBH) (m), Σ dbh= sum of diameter at breast height (1.3 m), N = number of trees

Basal Area

$$BA=\frac{\pi{dbh}^{2}}{40000}$$

where: BA = basal area of tree (m^2^), dbh = diameter at breast height (m), and π = pi (3.142).

Stand Volume

$V=\left( \pi dbh \times h \times0.7 \right)$/4

where: V = stand volume (m^3^/Ha), π = pi (3.142), dbh= diameter at breast height (m), h= tree height (m), form factor= 0.7.

Stand Density

$$SPH=\frac{N}{A\times10000}$$

where: SPH = Stand density per hectare, N = number of trees, A = plot area.

*
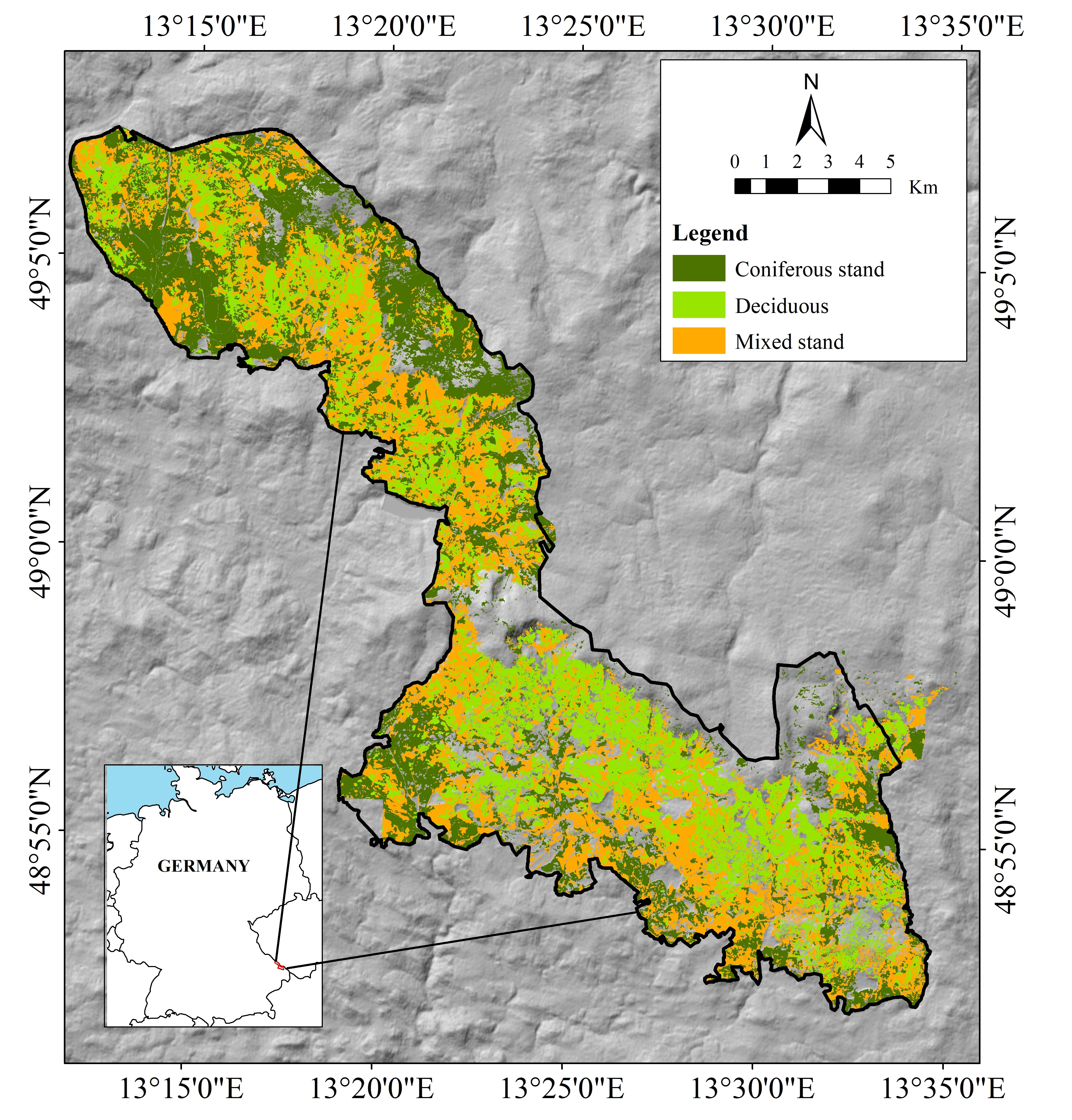
***Supplementary S4.** Forest Type Distribution Map in Bavarian Forest National Park. The coniferous type is commonly dominated by Norway spruce, the deciduous type is dominated by European beech, and the mixed stand consists of Norway spruce or silver fir and European beech. The map was created based on Land Cover Map of BFNP developed by Silveyra Gonzalez et al. ( 2018).

**References**

Atkins, J. W., Fahey, R. T., Hardiman, B. H., & Gough, C. M. (2018). Forest Canopy Structural Complexity and Light Absorption Relationships at the Subcontinental Scale. *Journal of Geophysical Research: Biogeosciences*, *123*, 1387–1405. https://doi.org/10.1002/2017JG004256

Bouvier, M., Durrieu, S., Fournier, R. A., & Renaud, J. P. (2015). Generalizing predictive models of forest inventory attributes using an area-based approach with airborne LiDAR data. *Remote Sensing of Environment*, *156*, 322–334. https://doi.org/10.1016/j.rse.2014.10.004

Hardiman, B. S., Bohrer, G., Gough, C. M., Vogel, C. S., & Curtis, P. S. (2011). The role of canopy structural complexity in wood net primary production of a maturing northern deciduous forest. *Ecology*, *92*(9), 1818–1827. https://doi.org/10.1890/10-2192.1

Kamoske, A. G., Dahlin, K. M., Stark, S. C., & Serbin, S. P. (2019). Leaf area density from airborne LiDAR: Comparing sensors and resolutions in a temperate broadleaf forest ecosystem. *Forest Ecology and Management*, *433*, 364–375. https://doi.org/10.1016/j.foreco.2018.11.017

Kane, van R., McGaughey, R. J., Bakker, J. D., Gersonde, R. F., Lutz, J. A., & Franklin, J. F. (2010). Comparisons between field- and LiDAR-based measures of stand structural complexity. *Canadian Journal of Forest Research*, *40*(4), 761–773. https://doi.org/10.1139/X10-024

Parker, G. G., & Russ, M. E. (2004). The canopy surface and stand development: Assessing forest canopy structure and complexity with near-surface altimetry. *Forest Ecology and Management*, *189*, 307–315. https://doi.org/10.1016/j.foreco.2003.09.001

Silveyra Gonzalez, R., Latifi, H., Weinacker, H., Dees, M., Koch, B., & Heurich, M. (2018). Integrating LiDAR and high-resolution imagery for object-based mapping of forest habitats in a heterogeneous temperate forest landscape. *International Journal of Remote Sensing*, *39*(23), 8859–8884. https://doi.org/10.1080/01431161.2018.1500071

van Ewijk, K. Y., Treitz, P. M., & Scott, N. A. (2011). Characterizing forest succession in central Ontario using lidar-derived indices. *Photogrammetric Engineering and Remote Sensing*, *77*(3), 261–269. https://doi.org/10.14358/PERS.77.3.261
